# Dietary patterns and the risk of Alzheimer’s disease in an elderly Iranian population: a case–control study

**DOI:** 10.1186/s41043-023-00398-y

**Published:** 2023-06-15

**Authors:** Rezvan Hashemi, Zahra Vahabi, Hamid Rasekhi, Farideh Shiraseb, Maryam Amini

**Affiliations:** 1 Department of Geriatric Medicine, Ziaeian Hospital, Tehran University of Medical Sciences, Tehran, Iran; 2 Geriatric Department, Tehran University of Medical Sciences, Tehran, Iran; 3 Neurology Division, CNS Department, Western University, London, ON Canada; 4 Department of Nutrition Research, National Nutrition and Food Technology Research Institute and Faculty of Nutrition Sciences and Food Technology, Shahid Beheshti University of Medical Sciences, Tehran, Iran; 5 Department of Community Nutrition, School of Nutritional Sciences and Dietetics, Tehran University of Medical Sciences, Tehran, Iran

**Keywords:** Alzheimer’s, Food patterns, Dementia, Elderly, Nutrition

## Abstract

**Background:**

An increasing number of studies in Western countries have shown that healthy eating patterns have a protective effect against cognitive decline and dementia, however, information about this relationship among non-western populations with different cultural environments is scarce. The present study investigated the association between dietary patterns (DPs) and cognitive function in the Iranian elderly.

**Methods:**

In this case–control study, the data of 290 elderly people in two groups of case and control (Mean age in case: 74.2 ± 8.6, in control: 67.3 ± 7.3 year) were analyzed. Two DPs of healthy and unhealthy were extracted from a 142-item dish-based food frequency questionnaire, and patterns driven by principal components analysis (PCA) of 25 food groups. Multivariate binary logistic regression calculated the odds ratio (OR) of cognitive impairment with adjustment for potential confounding factors.

**Results:**

A healthy DP, characterized by high consumption of fruits and vegetables, legumes, and nuts, was related to a decrease in the odds of Alzheimer’s disease in Iranian elderly people. Also, moderate adherence to an unhealthy food pattern was associated with an increase in the probability of the disease; however, the association was not statistically significant.

**Conclusion:**

In this elderly population, a healthy eating pattern was associated with reducing the risk of Alzheimer’s disease. Further prospective studies are recommended.

## Background

The population over the age of 60 will double by 2050 and the number of persons aged 80 years or older is expected to reach 426 million [1]. Delays in addressing the health needs and quality of life among this population will lead to irreparable complications. Dementia and Alzheimer’s disease are among the common diseases of this period of life which affect not only the life of the patients but also the life of the patient’s family members [2]. Dementia is a progressive chronic neurodegenerative disease and the most common type of dementia is one caused by Alzheimer’s disease. More than half of elderlies with dementia are suffering from this type of disease [3].

According to a report by the World Alzheimer’s Organization in 2020, 55 million people in the world were estimated to live with dementia, and every 3 s a new case of dementia occurs. Thus, it is predicted that by 2050, the number of sufferers will reach 139 million [4]

Alzheimer’s disease is a global concern that imposes a great financial burden on the patient and the medical care system of the communities [5]. Hence, in addition to pharmaceutical and non-pharmacological treatments, researchers pay special attention to ways to prevent or slow down the progress of dementia and Alzheimer’s disease at the beginning [6].

Alzheimer’s disease, the most common type of dementia in the elderly, is caused by the accumulation of beta-amyloid in the brain [7]. Beta-amyloid protein is deposited inside and between neurons and by causing inflammation and oxidative stress reaction, it causes neurodegeneration in the hippocampus, cerebral cortex, reduction of synaptic flexibility, and impaired nerve function [8–10]

This process in the brain is related to the activation of the immune system [11]. This disease causes impairment in recent or short-term memory and reduced learning and by the progression of the disease finally, the patients lose understanding and attention toward themselves.

Age and genetic predisposition have been known as unmodifiable and physical inactivity, blood pressure, type 2 diabetes, obesity, and smoking as modifiable risk factors for dementia.

The relationship between lifestyle and nutrition with dementia is well understood and it is documented that their modification at the beginning of the disease may delay or prevent dementia and Alzheimer’s disease [12]. However, the etiology of most types of dementia has not been completely known.

Dietary intake, in terms of its potential to prevent disease development, has been widely discussed. Although some epidemiological studies have reported that consumption of certain types of foods such as fish and vegetables may be preventive against dementia and Alzheimer’s disease, the results are still mixed.

Studies related to the effect of food on health have been conducted by two different approaches in which either the effect of the nutrients are investigated alone or the total food intake as a pattern is considered. In the first approach, the interactions of nutrients with each other are ignored, whereas from another point of view, all the interactions between nutrients are examined and DPs are identified [13–15].

Recent studies on the relationship between diet and dementia among the elderly have shifted from the role of nutrients or foods alone to the role of DPs which reflect the complexity of diet and eating behaviors.

So far, various studies have been conducted to assess the relationship between DPs with the occurrence of dementia worldwide [16, 17], and several studies have acknowledged the protective and positive effect of the Mediterranean diet on dementia and Alzheimer’s [18, 19]. However, no research has revealed any relationship between other DPs and dementia or Alzheimer’s. The present case–control study aimed to investigate the relationship between Iranian dietary patterns and occurrences of Alzheimer’s among the elderly.

## Methods

The current paper has been organized according to the STROBE checklist.

### Design and participants

It was an individually matched case–control study. The participants were the elderly who were referred to the geriatric clinics of Ziaeian and Roozbeh hospitals in Tehran. Patients who were with Alzheimer’s according to the criteria of the National Institute of Aging–Alzheimer’s Association 2011 (NIA–AA) [20], were older than 60 years and communicating with them or their companion was possible were included in the case group. The participants in the control group were also over 60 years, selected from the same hospitals, and did not have Alzheimer’s disease or any other cognitive disorders such as other non-Alzheimer’s dementias. Finally, 290 (152 cases, 138 control) people were recruited in the study.

### Ethical considerations

All participants provided written informed consent prior to the research. The study protocol was approved by the Ethics Committee of Tehran University of Medical Sciences which follows the Helsinki Declaration [21]. The ethics code was IR.TUMS.MEDICINE.REC.1400.893. November.10th. 2021.

### Assessment of covariates

Data of demographic information (age, gender, education, ethnicity, and marital status), medical history (any record of blood pressure, heart diseases, neurology, or mental disorders), medication, receiving nutritional supplements during three last month, and lifestyle (smoking, alcohol consumption) were collected. We measured the participants’ body weight (kg) and height (cm) using a Seca digital floor scale (500 gr precision) and a Seca stadiometer (0.5 cm precision), respectively. Weight and height were measured while they were fasting, standing, wearing light clothing, and no shoes.

### Assessment of dietary intake

The information on food intake, for both case and control groups, was collected by a semi-quantitative 142-item dish-based FFQ whose reliability and validity were evaluated, previously [22]. The questionnaires were completed and analyzed electronically. Consumed food items were categorized into 25 food groups based on the similarity of their nutrients, the opinion of the researchers, and previous studies (Table 1). In case the content of the nutrients in a food item was essentially different from other food items, or in case the consumption of that food item indicated a special eating habit, a separate food group was assigned to that(e.g., tea and coffee, salt, and mayonnaise). Due to memory impairment, patients with Alzheimer’s hardly recall the components of mixed foods they consumed. Since the ingredients of the mixed foods were embedded in the FFQ we used, the patients did not need to remember the frequency and amount of each component [23].

**Table 1:** Food groups used in the dietary pattern analysis

Food groups	Food items
Fast foods	Sausage, bolonga, hamburger, pizza
Red meat	Beef, veal, lamb, mince meat
Poultry & fish	Chicken, canned tuna, any type of fish
Eggs	Egg
Low fat dairy	Buttermilk, low-fat milk, plain yogurt
High fat dairy	Whole milk, whole milk yogurt, cocoa milk, condensed yogurt, cream, ice creams(traditional and non-traditional), cheese, cream cheese, Kashk
Coffee and tea	Coffee, tea
Fruits, fruit juices, and dried fruits	Dried and fresh Apricots, cantaloupe, tangerines, cherries, persimmons, oranges, fresh and dried peaches, apples, grapes, bananas, watermelons, melons, kiwis, strawberries, pomegranates, lemons, limes, fresh and dried berries, fresh and dried figs, pears, nectars, grapefruit, plums (yellow and red), Cantaloupe juice, apple juice, orange juice, lemon juice, dates, raisins, other dried fruits
Synthetic fruit juice	Packaged fruit juice, cola and other soft drinks
Vegetable	Lettuce, zucchini, carrots, cabbage, belly pepper, spinach, turnips, cucumber, eggplant, celery, garlic, onion, mushroom, green pepper, tomatoes, green leafy vegetables
Starchy vegetable	Corn, maize, green peas, green beans
Legumes	Lentils, beans, chickpeas, split peas, mung beans, soybeans
Whole grains	Oatmeal or cooked grits
Refined grains	Bread (Lavash, Barbari, Sengak, Baguette, Tufton), pasta, rice, noodles, Reshteh (Persian noodle), vermicelli
Potato	Baked potatoes, French fries
Nuts	Peanuts, almonds, pistachios, hazelnuts, walnuts, seeds
Olives	Green olives, olive oil
Sweets	Chocolate, cakes, cookies, creamy pastries, Persian Halva
Snacks	Biscuits, crackers, cheese curls, chips
Solid fats	Butter, margarine, animal oil, solid vegetable oil, mayonnaise
Vegetable oil	Liquid oils other than olive
Sugar and sweet substances	Sugar, Noghle, Shekarpanir, Nabat, candies, Gaz, Sohan, Halva shikari, honey, All kinds of jams, fruit compote
Spices and seasonings	All kinds of spices, ketchup
Salt	Salt
Pickles	Different kinds of pickles

### Statistical analysis

DPs were determined by PCA. To explore the dominant DPs, food groups were identified. To obtain a simple matrix with better interpretability and the extraction of the dominant food patterns, Varimax rotation was used. Food groups with factor loadings > 0.2 were considered important contributors to a dietary pattern.

To identify whether a factor should be retained, the study factors were naturally interpreted in conjunction with eigenvalues that was higher than 1 and the scree plot was determined. The factor score for each person was calculated by summing the intakes of food groups weighted by his/her factor loading. The derived factors (two DPs) were labeled as healthy and unhealthy based on our interpretation of the data and of the earlier literature. Tertiles of two DPs were defined to compare the lowest and highest tertiles of adherence to healthy and/or unhealthy DPs. To compare DPs with background variables, after determining the adherence score for each of the two DPs, the individuals' score was converted into low and high adherence.

Normality was tested by Kolmogorov–Smirnov. To compare means, an independent sample t-test was used. The Chi-square test was used to examine the relationship between the categorical variables. Regression logistics was used to investigate the relationship between tertiles of DPs (independent variable) with Alzheimer’s disease (dependent variable) and modify the effect of background variables namely gender, body mass index, age, and smoking. The first tertile of the dietary patterns score was considered as a reference. The results of the study were analyzed by SPSS version 25 software. Values less than 0.05 were considered significant for all tests.

## Results

The mean age of the study participants was 72.9 and their ages ranged between 62 and 95 years.

Most of the participants (46.2%) were of Turk ethnicity and the majority (41%) lived with their spouses. Thirty-two percent of the participants were illiterate and the education level of 36.6% was a diploma or higher. About 4% of the elderly reported using narcotics. As shown in Table 2, regarding the lifestyle, education, and age two groups were significantly different.

**Table 2:** Characteristics of case and control groups

Variables	Case (*n* = 152) No (%)	Control (*n* = 138) No (%)	*P*-Value
*Gender*			
Male	83 (52)	77 (48)	0.9
Female	68 (53)	61 (47)	
*Ethnicity*			
Fars	64 (48.5)	68 (51.5)	0.063
Turk	74 (55.2)	60 (44.8)	
Lur	4 (44.4)	5 (55.6)	
Kurdish	0	3 (100)	
Gilak	9 (81.8)	2 (18.2)	
*Life style*			
Single	29 (60.4)	19 (39.6)	0.00
Living with spouse	56 (40.8)	63 (59.2)	
Living with offspring(s)	38 (79.2)	10 (20.8)	
Living with caretaker	3 (100)	0	
*Education*			
Illiterate	56 (73.7)	20 (26.3)	0.00
Reading and writing	56 (52.3)	51 (47.7)	
Diploma and higher	39 (36.8)	67 (63.2)	
*Disease history*			
CVD*	38 (49.4)	32 (50.6)	0.8
Hyperlipidemia	2 (66.6)	1 (33.3)	
Renal diseases	2 (50)	2 (50)	
Age (years) Mean ± SD	74.2 ± 8.6	67.3 ± 7.3	0.00
Weight (kg) Mean ± SD	67.5 ± 13.4	71 ± 14.5	0.34
Height (cm) Mean ± SD	162.1 ± 9.1	162.9 ± 17	0.63
Body mass index (BMI) (kg/m^2^) Mean ± SD	25.7 ± 4.8	26.6 ± 4.9	0.10
Years of smoking Mean ± SD	4.2 ± 13.2	1.6 ± 7.2	0.033
Cigarettes per year Mean ± SD	0.2 ± 0.7	0.2 ± 0.8	0.64

*CVD Cardiovascular disease

Table 3 shows the factor loading obtained after varimax rotation. The first factor which accounted for 38.52% of the total variance (45.6) and had high loadings for salt, solid fats, red meat, starchy vegetables, poultry/fish, potatoes, green vegetables, pickles, mayonnaise, saturated fats, whole cereals, sweets, fast foods, and whole dairy products was labeled “Unhealthy”. The second factor which explained 7.08 of the total variance loaded on poultry/fish, fruits, nuts, olive oil, and sea foods was named “Healthy”.

**Table 3:** Factor loading matrix for major DPs among the elderlies

Food groups	Unhealthy dietary pattern	Healthy dietary pattern
Salt	0.93	
Solid fats	0.93	
Red meat	0.9	
Starchy vegetables	0.9	
Poultry/Fish	0.87	0.21
Potatoes	0.79	
Green vegetables	0.78	
Pickles	0.7	
Eggs	0.67	
Synthetic juice	0.67	
Mayonnaise	0.66	− 0.25
Saturated fats	0.57	
Whole grains	0.56	
Sweets	0.51	− 0.14
Fast foods	0.4	
High fat dairy foods	0.37	− 0.23
Fruits		0.73
Nuts		0.59
Refined grains	− 0.48	− 0.58
Tea		− 0.31
Olive oil		0.3
Sea foods	− 0.16	0.18
% of variance	38.52	7.08

Values < 0.20 were excluded. Bartlett’s Test of Sphericity < 0.001. KMO = 0.897, Total variance: 45.6

As shown in Table 4, after adjusting for standard energy intake, age, gender,BMI, ethnicity, lifestyle, education, and smoking high adherence to the “Healthy DP” compared to low adherence, significantly lowered odds of Alzheimer’s disease by 0.62. In addition, moderate adherence to the “Unhealthy DP” in all models was related to higher odds of Alzheimer’s disease compared to low adherence; however, this difference was not statistically significant.

**Table 4:** Odds ratios of Alzheimer’s disease for different tertiles of DPs

Variables	OR	95% CI	P*	OR	95% CI	P**	OR	95% CI	P***	OR	95% CI	P****
*Healthy DP tertiles*												
Tertile I	1	–	–	1	–	–	1	–	–	1	–	–
Tertile II	1.5	0.85–2.7	0.15	1.5	0.81–2.8	0.19	0.97	0.45–2.11	0.95	0.97	0.45–2.11	0.94
Tertile III	0.63	0.315–1.12	0.14	0.62	0.32–1.2	0.15	0.38	0.16–0.87	0.02	0.38	0.16–0.87	0.02
*Unhealthy DP tertiles*												
Tertile I	1	–	–	1	–	–	1	–	–	1	–	–
Tertile II	1.05	0.6–1.8	0.87	1.4	0.41–4.8	0.54	1.14	0.3–4.4	0.85	1.125	0.29–4.4	0.86
Tertile III	0.63	0.36–1.12	0.11	0.88	0.42–1.8	0.75	0.89	0.36–2.24	0.81	0.87	0.35–2.2	0.77

*None of confounders were adjusted. **Adjusted by age, gender, and energy intake. ***Adjusted by standard energy intake, age, gender, ethnicity, life style, education, and smoking. ****Adjusted by standard energy intake, age, gender, BMI, ethnicity, life style, education, and smoking

## Discussion

In our study, two dominant DPs were identified. The risk of Alzheimer’s disease among the elderly population who followed a healthy DP that contained high amounts of nuts, fruits, olive oil, chicken, and low amounts of high-fat dairy products, mayonnaise, and tea was reduced. Moderate adherence to an unhealthy DP was associated with an increased risk of Alzheimer’s disease, although this difference was not statistically significant.

In an elderly French population, participants who had the highest adherence to a healthy dietary pattern characterized by high consumption of fruit, whole grains, fresh dairy products, and vegetables had better cognitive performance compared to those who had the least adherence [24]. A prospective cohort study of the US elderly showed that older adults with higher consumption of salad dressing, nuts, fish, tomatoes, chicken, cruciferous vegetables, fruits, and dark green leafy vegetables and lower intake of high-fat dairy products, red meat, organ meats, and butter had a lower risk of Alzheimer’s disease which is consistent with the results obtained in the current study [25].

In a cross-sectional study of an elderly Chinese population, among the individuals who had a “green fruit pattern” characterized by high consumption of fruits and vegetables, soy products, and legumes the risk of Alzheimer’s disease had been reduced [26].

Overall, consistent with other studies our study showed that fruits and vegetables can reduce the risk of cognitive impairment because of their high content of antioxidants, which are associated with a reduced risk of cognitive disorders [27]. Epidemiological and animal research also supports the protective effect of fruits and vegetables against cognitive disorders and dementia. The brain is highly susceptible to oxidative damage, and fruits and vegetables are rich in antioxidants [28]. In addition, according to animal studies, antioxidants prevent nerve damage and improve cognitive function [29].

Vitamin C is a strong water-soluble antioxidant that prevents the oxidative process by donating its electrons [30]. A study on mice models showed that the antioxidant vitamins in green leafy vegetables could be beneficial for cognitive health. It was also found that folate found in some fruits and vegetables is associated with cognitive function and dementia [31]. Folate deficiency can increase homocysteine levels, which have had direct neurological effects in animal studies [32, 33]. Therefore, the beneficial effect of antioxidants from the diet may be more important for older people whose antioxidant requirements are higher. There is accumulating evidence that indicates increased consumption of nuts, whole grains, milk, and dairy products, especially low-fat milk and low-fat dairy products, is associated with better cognitive performance and a reduced risk of Alzheimer’s disease [34, 35].

The healthy DP in the current study included high amounts of nuts which confirms this scientific fact. Furthermore, vitamin E which is abundant in nuts and fortified cereals has been shown to improve lifespan and mitochondrial and neurological function in aged mice [36].

The favorable effects of omega-3 polyunsaturated fatty acids (PUFA) of marine origin, including Eicosapentaenoic acid (EPA) and Docosahexaenoic acid (DHA), on cognitive decline or dementia, have been well established in several epidemiological studies [17, 37]. The effect can be attributed to the role of fatty acids in maintaining structural integrity, and nerve membrane, and determining the fluidity of synaptosomal membranes and consequently the regulation of neurotransmission. In addition, the protective effect of PUFA can be related to the anti-inflammatory and vascular protection effects exerted by these fatty acids [38].

Also, increasing evidence on the role of several B vitamins, in particular, there is folate, vitamins B6 and B12, which are required for proper DNA methylation and to prevent the accumulation of homocysteine, which potentially causes neurotoxic effects [37].

Very recent studies in Western countries have shown that dietary patterns rich in red meat and processed meat are associated with poor cognitive performance and faster cognitive decline [39, 40]. These findings are consistent with the results of the present study in which an unhealthy DP characterized by high consumption of red meat and saturated fat increased the risk of Alzheimer’s disease in moderate consumers.

To the best of the authors’ knowledge, there has been no similar study in Iran to have such a large sample size of patients with dementia. On the other hand, as mentioned earlier, for the first time in this study a 142-item dish-based FFQ was used to measure the food intake of the patients with dementia. Because of cognitive disorders among these patients, they poorly remember the ingredients of mixed foods and the consumption frequency of each ingredient. This tool, therefore, could collect data much easier, faster, and more accurately compared to item-based FFQs.

Our study had several limitations. It was a case–control study and we cannot rule out the possibility that the dietary habits of the participants may have changed with the onset of dementia symptoms as a result of various factors namely changes in the social environment, changes in smell, taste, physical activity, and drugs affecting weight. Therefore, the causal relationship between nutrition and cognitive performance cannot be determined in the present study and the results should be interpreted with caution. In this regard, prospective data are recommended for further clarification.

Serum levels of folate and vitamins were not investigated in our study. Meanwhile, the association of Alzheimer’s disease with some nutritional factors was not significant in this study. The reasons can be the simultaneous conduction of the project with the COVID-19 epidemic, lockdown, and people's reluctance to participate in the intervention, followed the smaller sample size and the possibility of Alzheimer’s disease overlapping with other types of dementia in the participants.

Due to the mixed results obtained from related studies, there is a need for extensive interventions with a larger sample size and a longer follow-up period to investigate the effect of different diets on Alzheimer’s disease in the elderly in Iran.

## Conclusion

The present study suggests that a healthy dietary pattern may have a preventive effect against Alzheimer’s disease in Iranian elderly people. Considering the fact that the elderly population in the country is increasing rapidly and taking into account the high prevalence of dementia among them, the results of this study can help authorities to provide more effective services to slow down the rate and reduce the burden of the disease in the country.

## Data Availability

The datasets used and/or analyzed during the current study are available from the corresponding author on reasonable request.

## Abbreviations

DPs: Dietary patterns
PCA: Principal components analysis
NIA–AA: National Institute of Aging–Alzheimer’s Association
FFQ: Food frequency questionnaire
BMI: Body mass index
PUFA: Poly unsaturated fatty acids
EPA: Eicosapentaenoic acid
DHA: Docosahexaenoic acid
